# Intracellular Expression of PAI-1 Specific Aptamers Alters Breast Cancer Cell Migration, Invasion and Angiogenesis

**DOI:** 10.1371/journal.pone.0164288

**Published:** 2016-10-18

**Authors:** Yolanda M. Fortenberry, Stephanie M. Brandal, Gilles Carpentier, Malvi Hemani, Arvind P. Pathak

**Affiliations:** 1 Department of Pediatric Hematology, The Johns Hopkins University School of Medicine, Baltimore, MD, United States of America; 2 Department of Biological Chemistry, The Johns Hopkins University School of Medicine, Baltimore, MD, United States of America; 3 Laboratoire CRRET, Faculté des Sciences et Technologie, Université Paris-Est Créteil, 61 avenue du général De Gaulle, 94010 Créteil, France; 4 Russell H. Morgan Department of Radiology and Radiological Science, The Johns Hopkins University School of Medicine, Baltimore, MD, United States of America; Columbia University, UNITED STATES

## Abstract

Plasminogen activator inhibitor-1 (PAI-1) is elevated in various cancers, where it has been shown to effect cell migration and invasion and angiogenesis. While, PAI-1 is a secreted protein, its intercellular levels are increased in cancer cells. Consequently, intracellular PAI-1 could contribute to cancer progression. While various small molecule inhibitors of PAI-1 are currently being investigated, none specifically target intracellular PAI-1. A class of inhibitors, termed aptamers, has been used effectively in several clinical applications. We previously generated RNA aptamers that target PAI-1 and demonstrated their ability to inhibit extracellular PAI-1. In the current study we explored the effect of these aptamers on intracellular PAI-1. We transiently transfected the PAI-1 specific aptamers into both MDA-MB-231 human breast cancer cells, and human umbilical vein endothelial cells (HUVECs) and studied their effects on cell migration, invasion and angiogenesis. Aptamer expressing MDA-MB-231 cells exhibited a decrease in cell migration and invasion. Additionally, intracellular PAI-1 and urokinase plasminogen activator (uPA) protein levels decreased, while the PAI-1/uPA complex increased. Moreover, a significant decrease in endothelial tube formation in HUVECs transfected with the aptamers was observed. In contrast, conditioned media from aptamer transfected MDA-MB-231 cells displayed a slight pro-angiogenic effect. Collectively, our study shows that expressing functional aptamers inside breast and endothelial cells is feasible and may exhibit therapeutic potential.

## Introduction

The association between the plasminogen activator system and cancer progression is well documented [1–4]. The major players in this system are the urokinase plasminogen activator (uPA), the uPA receptor (uPAR) and the uPA inhibitor, plasminogen activator inhibitor-1 (PAI-1). Increased tumor uPA expression is associated with a decrease in overall survival rate in individuals with early-stage breast cancer [5–7]. In addition, high concentrations of PAI-1 correlate with a poor prognosis (i.e. the “PAI-1 paradox”) in various gynecological cancers including breast and ovarian [8,9]. This finding is paradoxical since PAI-1 inhibits uPA, which in turn should inhibit or slow cancer progression. PAI-1 has been shown to regulate tumor cell adhesion, migration, invasion, and angiogenesis [9–11]. This is partly because of its interaction with the basement membrane protein, vitronectin [12,13]. Despite a plethora of data supporting PAI-1’s role in cancer, there is still controversy concerning its exact influence on cancer progression, as it has been shown to exhibit both pro- and anti-tumor effects.

The development of PAI-1 inhibitors as therapeutics has gained much ground over the past decade. Most PAI-1 inhibitors consist of monoclonal antibodies, peptides, low molecular weight compounds, and chemical suppressors [14,15]. Recently, a new class of nucleic acid molecules termed aptamers is receiving attention as potential therapeutic agents in cancer treatment [16]. Nucleic acid aptamers are short RNA or DNA molecules that bind to their target protein with high affinity and specificity. They are generated by using an in vitro selection method termed, SELEX (Systematic Evolution of Ligands by Exponential Enrichment). Aptamers have been developed to a variety of proteins including growth factors, receptor proteins, coagulation proteins, viruses, and many more [17–19]. We and others recently developed RNA molecules to PAI-1 to combat its activity by disrupting its ability to associate with vitronectin [20,21]. Additionally, these aptamers altered cell migration, adhesion and angiogenesis when administered exogenously [22]. In the current study, we investigated how these aptamers behave when expressed endogenously or within breast cancer and endothelial cells. Specifically, we assessed the effects of the PAI-1 specific aptamers on their ability to regulate human breast cancer cell adhesion, migration and invasion as well as angiogenesis. This study was designed to assess the differences between intracellular and extracellular aptamer expression in these cells. Consequently, it is a natural follow up to our original study demonstrating differences in intracellular aptamer expression [22]. We showed an aptamer dependent decrease in migration and invasion of breast cancer cells. The decrease correlated with an increased association of PAI-1 with uPA. Additionally, the intracellular aptamers caused a significant decrease in angiogenesis. Collectively, our results illustrate that aptamers are viable therapeutic agents not only when administered exogenously but also when expressed endogenously.

## Materials and Methods

### Cell Culture

The MDA-MB-231 human breast cancer cell line was obtained from the American Type Culture Collection (Manassas, VA). The cells were cultured in Dulbecco’s modified Eagle’s medium (DMEM) supplemented with 10% fetal bovine serum, and penicillin (100 units/ml), streptomycin (100 μg/ml). Human umbilical vein endothelial cells (HUVECs), purchased from Invitrogen (Carlsbad, CA), were cultured in endothelial cell media supplemented with 5% fetal bovine serum and endothelial cell growth supplement (ScienCell Research Laboratories, Carlsbad, CA). HUVECs at passages 3–7 were used in all experiments. All cells were maintained in a humidified chamber with 5% CO_2_ at 37°C.

### Transient Transfection

MDA-MB-231 cells were transiently transfected using Lipofectamine 2000 according to the manufacturer’s protocol (Invitrogen, Frederick MD). The HUVECs were transfected using the TransPass HUVEC Transfection Reagents (New England Biolab, Ipswick, MA). The cells were seeded in 6 well plates and incubated overnight or until they reached a confluent level of 70–90% in antibiotic free DMEM medium. The next day, 2.5 μl of Lipofectamine 2000 or 5 μl Trans Pass and 0–100 pmoles of RNA aptamer, diluted in Opti-MEM medium, were mixed gently and added to cells. Culture medium was changed after 6 hours post-transfection and then the cells were further incubated at 37°C in 5% CO_2_ for 24 hours in either DMEM with FBS or DMEM without FBS. The cells cultured in serum free medium were used in conditioned medium preparations. At 48 hours post-transfection the conditioned media from the cells incubated in serum-free was collected and the cells were discarded. The cells incubated in serum containing medium were detached, washed and counted for use in subsequent experiments.

### RNA aptamer in vitro transcription

The RNA aptamers (WT15, SM20, and Sel 2) were transcribed as detailed previously (20). The cDNAs were transcribed to RNA using a DuraScribe T7 transcription kit (Epicenter Biotechnologies, Madison WI). Briefly, 2 μg of linearized template DNA and the T7 promoter were incubated with 100 mM DTT, 50 mM ATP, GTP, 2’-F-dCTP, and 2’F-dUTP in the presence of 10 mM Durascribe T7 enzyme mix. The reaction was incubated at 37°C for 6 hours prior to adding DNase I (1 MBU) in order to remove the DNA template. The transcript was then extracted with phenol/chloroform/isoamyl alcohol. An equal volume of 2x formamide loading buffer was then added and incubated at 65°C for 5 minutes. The RNA transcript was cooled to room temperature and subjected to electrophoresis on a 12% 7M Urea denaturing gel. The RNA was visualized by UV shadowing, excised from the gel, minced, and incubated in 2 ml TE buffer overnight at 4°C. The next day, we removed the RNA and concentrated it using Amicon Ultra centrifugal filters (Millipore, Billerica, MA). The RNA concentration was determined and used in subsequent experiments. The RNA aptamers were incubated at 65–75°C for five minutes before being used in all experiments.

### Total RNA purification from the cells

Total RNA was isolated from both transfected and non-transfected cells. The cells were homogenized using QIA shedder spin columns according the manufacturer’s protocol (Qiagen, Valencia, CA USA). The buffer used to homogenize the cells contained denaturing guanidine-thiocyanate, which inactivates RNases; thereby, ensuring the purification of intact RNA. The RNA was then extracted and purified using the RNeasy Mini Kit (Qiagen) following the protocol established by the manufacturer. The final RNA product was eluted from the purification column into 30–50 μl dH_2_0. The RNA was transcribed into cDNA using the Promega kit (Promega, Madision WI, USA). Briefly, approximately 1 μg of isolated RNA was incubated with 10 mM dNTPs, RNasin (Promega), and M-MLV reverse transcriptase enzyme (Promega). The reaction was incubated at 37°C for 1 hour. The cDNAs were then subjected to PCR using the following primer for each respective gene; **PAI-1** 5’: aat cag acg gca gca ctg tc and 3’: ctg aac atg tcg gtc att cc; **uPA**-5’: ggc agc aat gaa ctt cat caa gtt cc and 3’: tat ttc caca gtg ctg ccc tcc g; **uPAR**-5’: gag ggg gat ttc agg ttt agg, and 3’: aca gga gct gcc ctc gcg ac: β-actin– 5’ atc tgg cac caca cc ttc tac aat ga, and 3’ cgt cat act cct gct tgc tga tcc ac. The cDNAs were amplified with each cycle consisting of a 30 second denaturing step at 94°C, a 30 second annealing step at 50–60°C, depending on the primer set, and a 30 second elongation step at 72°C. The pre amplification step was performed at 94°C for 5 minutes and the post-amplification step was at 72°C for 5 minutes. The RNA expression of the aptamers were determined by using the primers to the ‘fixed’ regions of the aptamers [20].

### Western Blot analysis

Cell lysates from transfected cells were concentrated and the protein concentration was determined by the Bio-Rad protein assay kit (Bio-Rad, Hercules, CA). For cell lysates, the transfected cells were washed twice in cold 1X PBS buffer. This was followed by adding RIPA buffer and incubating on ice for 15 minutes. The cells were then scraped off the dish using a cell scraper and the cell suspension was centrifuged from 5 minutes at 14,000 rpm. Approximately 21 μg of total protein was separated on a 10% SDS-PAGE gel and electro-transferred onto nitrocellulose membranes. The membranes were probed with the following primary antibodies overnight at 4°C, respectively; rabbit-anti human PAI-1 affinity purified antibody, and rabbit anit-human uPA affinity purified antibody (Molecular Innovations, Novi, MI). The following day, the primary antibodies were removed, the membranes were washed 3X at room temperature, and then incubated for 1 hr at room temperature with the appropriate horseradish peroxidase-conjugated secondary antibody. The proteins were visualized by the ECL kit (Amersham Bioscience, Pittsburgh, PA).

### Cell Migration and Invasion Assays

The migration assays were carried out in a modified Boyden Chamber system as described previously [22](Corning INC, Corning, NY USA). The migration assays were performed on BD culture inserts containing an 8-μm diameter pore size membrane. The invasion assays were carried out using CHEMICON cell invasion assays kit (Chemicon International, Billerica, MA USA). The chambers were hydrated with 300 μl serum free media at 37°C in 5% CO_2_ for 1–2 hours prior to use. This permitted rehydration of the ECM layer, which is a reconstituted basement membrane matrix of proteins derived from the Engelbreth Holm-Swarm (EHS) mouse tumor (Chemicon International, Billerica, MA USA). The cells were detached from the plate, washed, and 0.5x10^6^ cells were re-suspended in 300 μl of fresh serum free media. The cells were then seeded in triplicates in the upper chamber of the 12 well transwells plate (8 μM pore). The lower chamber of the wells contained 500 μl of DMEM supplemented with 10% FBS. The cells were allowed to migrate or invade at 37°C for 24–72 hours. After this incubation period, the inserts were removed and the non-migratory cells on the top surface of the filters were gently removed by using a cotton swab. This step was repeated twice. For both the invasion and migration assays, 500 μL of the staining solution was added to the unoccupied wells of the plate, and the cells on the lower surface of the membrane were stained by dipping the inserts into the staining solution for 20 minutes. This was followed by dipping the inserts into water several times to rinse. The inserts were air dried and then placed in 10% acetic acid to dissolve the incorporated dye. Approximately 50 μl of the dye/solute mixture was then added to a 96-well plate and read at an OD of 560 nm using a VersaMax Plate Reader (Molecular Devices, Sunnyvale, CA, USA).

### Cell adhesion Assay

Cells were seeded at a concentration of 5X10^4^ in 96 well vitronectin coated plates (5 μg/ml). The cells were allowed to adhere for 1 hour at 37°C. Non-adherent cells were removed and the plates were washed twice with warm PBS. The number of attached cells was determined by MTT (3-(4, 5-dimethylthiazol-2-yl)-2,5-diphenyltetrazolium bromide) assay. MTT (5mg/ml) was suspended in PBS and 10% MTT solution was prepared in serum free medium. The MTT solution was added to the adherent cells and incubated at 37°C for 2 hours. The MTT solution was gently removed and 200 μl DMSO was then added to each well, and incubated for 15 minutes at 37°C. The absorbance was measured using a VersaMax microtiter plate reader at 600 nm.

### Cell Viability Assay

To assess cell viability after transfection, approximately 5000 cells were plated in triplicates in a 96 well dish after 24 hours post transfection. The cells were assessed for growth by the MTT assay as described above at various time points. Time zero was the first day 0 hours (24 post transfection), 24 hours (48 hour post transfection), and 48 hours (72 hours post transfection).

### uPA Activity assay

An indirect uPA activity assay was performed using a synthetic uPA substrate to measure uPA activity. Briefly, 21 μg of cell lysates or 50–100 μl of conditioned media from transfected and non-transfected cells were added to buffer containing uPA substrate (S-2444, Chromogenix, West Chester, OH). All assays were performed in BSA coated plates. Triplicate samples were incubated for 30 min at 37°C and the reaction was monitored spectrophotometrically. The data presented are the average of 2 separate experiments and represents the amount of uPA activity remaining compared to the untreated control cells.

### Angiogenic Cytokine Analysis

To assess the protein levels of angiogenic cytokines secreted into the conditioned media of transfected and non-transfected cells, we used a commercially available human angiogenesis ELISA cytokine profiling kit (Signosis, Santa Clara, CA). Approximately 50 μl of conditioned medium was added to wells containing a primary antibody against specific cytokines. The samples were incubated for 1–2 hours at room temperature with gentle shaking. The sample solution was then aspirated and the wells were washed three times with 1× assay wash buffer prior to adding 100 μl of biotin-labeled antibody mixture. The antibody mixture was incubated for 1 hour at room temperature with gentle shaking. The antibody was then removed; the wells were washed three times with 1× assay wash buffer; then streptavidin-HRP conjugate solution was added to each well, and incubated for 45 minutes at room temperature with gentle shaking. Prior to adding the substrate, the wells were washed three times with 1× assay wash buffer. The reaction was stopped after 30 minutes, and the optical density was determined using a microplate reader at 450 nm.

### Endothelial Tube Formation or Angiogenesis Assay

Matrigel (BD Biosciences, San Jose CA, USA) was added to the wells of a 15-well treated microscope angiogenesis u-slide (Ibid, Martinsried, Germany) in a volume of 10 μl and allowed to solidify at 37°C for 30 min. After the Matrigel solidified, 1.5× 10^4^ human umbilical vein endothelial cells (HUVECs) (transfected and non-transfected) were added in 50 μl of DMEM supplemented with 10% FBS. The cells were incubated at 37°C with humidified 95% air/5% CO_2_ for 18 h normal HUVEC growth media. In the co-cultured experiments, the conditioned media from transfected and non-transfected MDA-MB-231 cells were collected at 72 hour post-transfection. HUVECs (non-transfected) were grown in the presence of CM from aptamer transfected MDA-MB-231 cells (0–100 pmol). The HUVECs were then harvested, plated on matrigel, and tube formation was assessed. The tubes (cells) were labeled with Calcein AM Fluorescent Dye (8 μg/ml; BD Biosciences, San Jose, CA) for 30–45 minutes at 37°C, 5% CO_2_, and photographed using a Nikon TS100 fluorescent microscope (Melville, NY) at a 4× magnification. Four independent fields were acquired from each slide and the morphological aspects of the tube network quantified using the angiogenesis analyzer plugin [Gilles Carpentier. ImageJ contribution: Angiogenesis Analyzer. ImageJ News, 5 October 2012.] for ImageJ [Schneider, C.A., Rasband, W.S., Eliceiri, K.W. "NIH Image to ImageJ: 25 years of image analysis". [23]. This plugin, customized for the present work, enabled the analysis of the vascular organization of HUVECs derived tube network or mesh. Morphological parameters that were extracted from images of the HUVEC derived tube network included the mesh index (i.e. the mean distance separating two master junctions in the network), mesh size (i.e. the mean mesh size), mean total branch length, mean total branching length (i.e. sum of length of the trees composed from segments and branches), mean total master segment length (i.e. sum of the length of the detected master segments), mean total segment length (i.e. sum of length of the segments) and the mean total length (i.e. sum of length of segments, isolated elements and branches). Definitions for each of these terms can be found in S1A Fig. To determine if the different aptamers significantly affected endothelial tube formation we employed a repeated measures analysis of variance using the aptamer type and experimental condition as ‘between factor’ variables and the experimental repeat as the ‘within factor’ or ‘repeated’ variable. All data were analyzed using the NCSS software package (Kaysville, Utah).

### Statistical analysis

Data are presented as mean values with standard deviation (SDM). Significance among the groups relative to ‘no aptamer’ control groups was tested using an unpaired Student’s *t* test. The test was calculated using GraphPad Prizsm software (p values <0.05 were considered statistically significant).

## Results

### Endogenous expression of PAI-1 specific RNA aptamers

**Fig 1:**
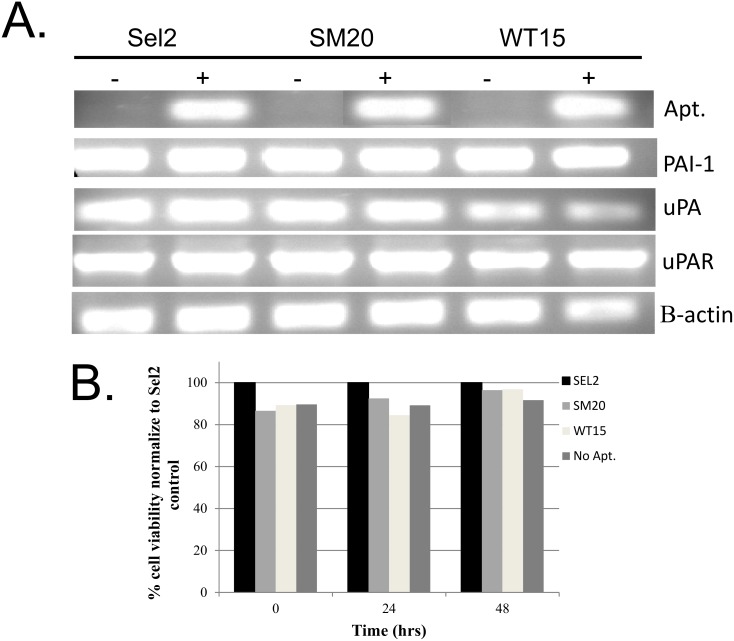
Expression of RNA aptamers in MDA-MB-231 cells. (**A**) MDA-MB-231 cells were transfected with the aptamers (Sel2, SM20, and WT15). Total RNA was isolated from transfected (+) and non-transfected (-) cells, then RT-PCR analysis were performed. Expression of the aptamer, PAI-1, uPA, uPAR, and β-actin is shown. This experiment was repeated at least three independent times with similar results. (**B**) The transfected and non-transfected cells were plated in a 96-well dish and allowed to grow in serum containing media for 72 hours post-transfection. The first time point (0) was assessed at 24 hours post transfection. An MTT assay was performed 24 hour intervals. The data was normalized to control cells (set at 100%) at each given time point. Each data point was performed in triplicate. The experiments were repeated at least twice with similar results.

The highly invasive and metastatic human MDA-MB-231 breast cancer cells, which express elevated levels of PAI-1 were used in these studies. The aptamers (SM20, WT15, and the control aptamer, Sel2) were transiently transfected into the MDA-MB-231 cells as detailed in the Materials and Methods. As illustrated in Fig 1, all three aptamers were strongly expressed, relative to non-transfected MDA-MB-231 cells. The non-transfected cells were subjected to the same transfection conditions as the transfected cells. To ensure that an equal amount of RNA was loaded, we gauged the expression of β-actin, which was similar in all experimental groups (Fig 1A). Accordingly, increases in aptamer expression were a direct result of the transfected RNA and not total RNA concentrations.

We next assessed whether the transfected aptamers alter the RNA expression levels of uPA, uPAR, and PAI-1, as each of these plays a vital role in the migratory and invasive potential of cancer cells [1,24]. We did not observe any significant variation in the expression levels of any of these genes relative to non-transfected MDA-MB-231 cells (Fig 1A). A minor decrease in uPA expression was noticed in cells transfected with WT-15 (Fig 1A); however, considering that β-actin was also low, this was most likely due to the RNA load as opposed to the transfected aptamers. In subsequent repeated experiments, we confirmed that the uPA expression was not altered in these cells (data not shown). Based on these results, we concluded that the intracellular expression of the aptamers did not appreciably alter the RNA expression of PAI-1 or its downstream effectors.

Considering that nucleic acids can potentially cause cell death when transfected, we next determined the toxicity of the aptamers to MDA-MB-231 cells by performing an MTT assay at 24 hour intervals. Fig 1B shows that cell viability was maintained over the 48 hour period compared to the control aptamer, indicating that the aptamers were not toxic to the cells. Cells transfected with the aptamers displayed a slight decrease in cell viability compared to control; however, this difference was not significant. From these results, we can infer that the neither the PAI-1 aptamers nor the control aptamer had an impact on cell proliferation.

### The aptamers accelerate the inhibitory potential of PAI-1 towards uPA

**Fig 2:**
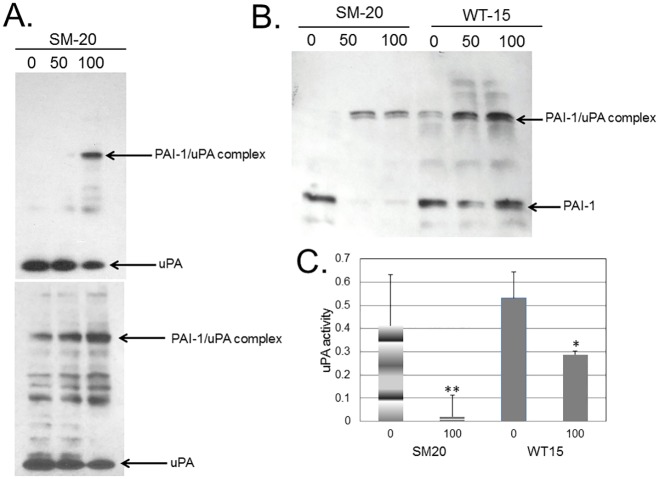
Intracellular aptamers inhibit uPA activity. **(A)** Intracellular uPA or (**B**) PAI-1 protein levels in cellular extracts of transfected and non-transfected MDA-MB-231 cells were analyzed by Western blot using an antibody to either uPA (A) or PAI-1 (**B**). (**A**) Top panel (short exposure) and the lower panel (longer exposure). Total protein concentration was determined and 21 μg protein was added at each experimental condition. The upper band corresponds to the PAI-1/uPA complex (**A-B**). (**C**) Intracellular uPA activity was determined in cellular extracts using a chromogenic assay in non-transfected cells (0) and cells transfected with 100 pmol RNA aptamers. Each experiment was performed at least three times with comparable results. **p<0.01, *p<0.05.

Next we assessed if the transfected aptamers altered the protein expression of PAI-1, uPA and uPAR in MDA-MB-231 cells (Fig 2). The SM20 aptamer elicited a concentration dependent decrease in uPA protein levels (Fig 2A). Similar results were obtained from cells transfected with WT15 (data not shown). The aptamer dependent decrease in ‘unbound’ uPA protein corresponded to an increase in the PAI-1/uPA complex (Fig 2A). In cells transfected with 100 pmol SM20, the immunoreactive band corresponding to the PAI-1/uPA complex was significantly stronger compared to non-transfected cells. However, upon longer exposure, a noticeable aptamer-dependent increase in the PAI-1/uPA complex was seen (Fig 2A; **bottom**). The PAI-1 protein decreased in cells transfected with SM20 and WT15; however, the effect in cells transfected with WT15 was less pronounced (Fig 2B). Interestingly, we noticed a more decreased PAI-1 protein expression in cells transfected with 50 pmol WT15, as compared to the 100 pmol treatment. Nevertheless, since this decrease was not significant, we chose the 100 pmol treatment for subsequent experiments. This allowed us to directly compare the two aptamers at the same concentration. Similar to uPA, an increase in aptamer concentration correlated with an increase in the PAI-1/uPA complex and a decrease in PAI-1 protein (Fig 2A and 2B). The protein expression of uPAR was unchanged (not shown). Hence, these results suggest that the aptamers caused an increase in PAI-1’s intracellular association with uPA.

**Fig 3:**
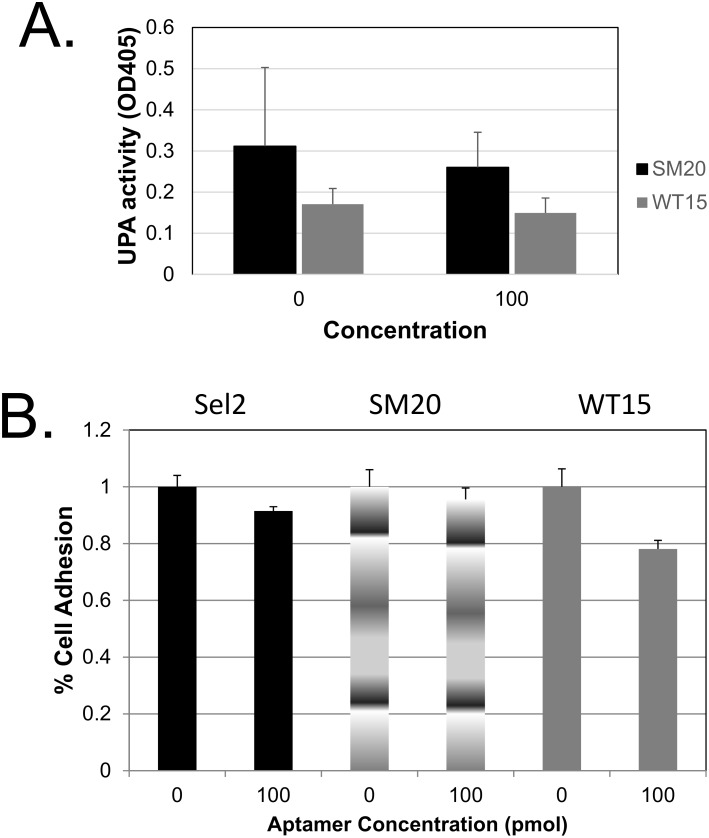
Effects of the RNA aptamers secreted uPA activity and on adhesion of MDA-MB-231 cells to vitronectin. **(A)** Conditioned medium from MDA-MD-231 cells was collected and assayed for uPA activity as detailed in the Materials and Methods section. **(B)** MDA-MB-231 cells transfected with aptamers (Sel2, SM20, and WT15) or non-transfected cells were added to vitronectin coated plates and incubated for 1 hour at 37°C. The non-adherent cells were removed and the adherent cells were assessed by an MTT assay analysis. The percent of adherent cells were normalized to the percent of cells adhering in the absence of aptamers. All reactions were done in triplicates and repeated at least three times; error bars represent the standard deviation of the data. No significant difference was observed in any on the treatment groups compared to non-transfected cells.

We then evaluated intracellular uPA’s activity in the transfected cells using a commercially available chromogenic substrate. The uPA activity decreased in cells transfected with both aptamers when compared to non-transfected cells (Fig 2C). Additionally, we observed a decrease in secreted uPA activity in the conditioned media of these cells (Fig 3A); however, the decrease was not significant. Consequently, we hypothesize that the intracellular aptamers cause an increase in the inhibitory potential of PAI-1 towards uPA by enhancing PAI-1’s ability to or the rate at which PAI-1 associate with uPA.

### Adhesion to vitronectin (VN) is not significantly altered in aptamer expressing breast cancer cells

We then assessed the ability of the transfected cells to adhere to vitronectin. There was a slight decrease in adhesion in cells expressing the control aptamer as well as SM20. In contrast, the aptamer, WT15 caused a more profound decrease in cell adhesion to vitronectin (Fig 3B). These data imply that the SM20 does not alter the ability of breast cancer cells to adhere to vitronectin; however, WT15 appears to have a greater, but not significant, effect on adhesion of MDA-MB-231 cells to vitronectin. In our experiment we used trypsin to detach the cells. Since using trypsin to detach cells could potentially impede the ability of the cells to adhere to vitronectin, we repeated this experiment with a 1 mM EDTA solution instead of trypsin and gentle rocking to detach the cells. We obtained similar results using both methods (not shown).

### Cell migration and invasion are both decreased in breast cancer cells expressing the aptamers

**Fig 4:**
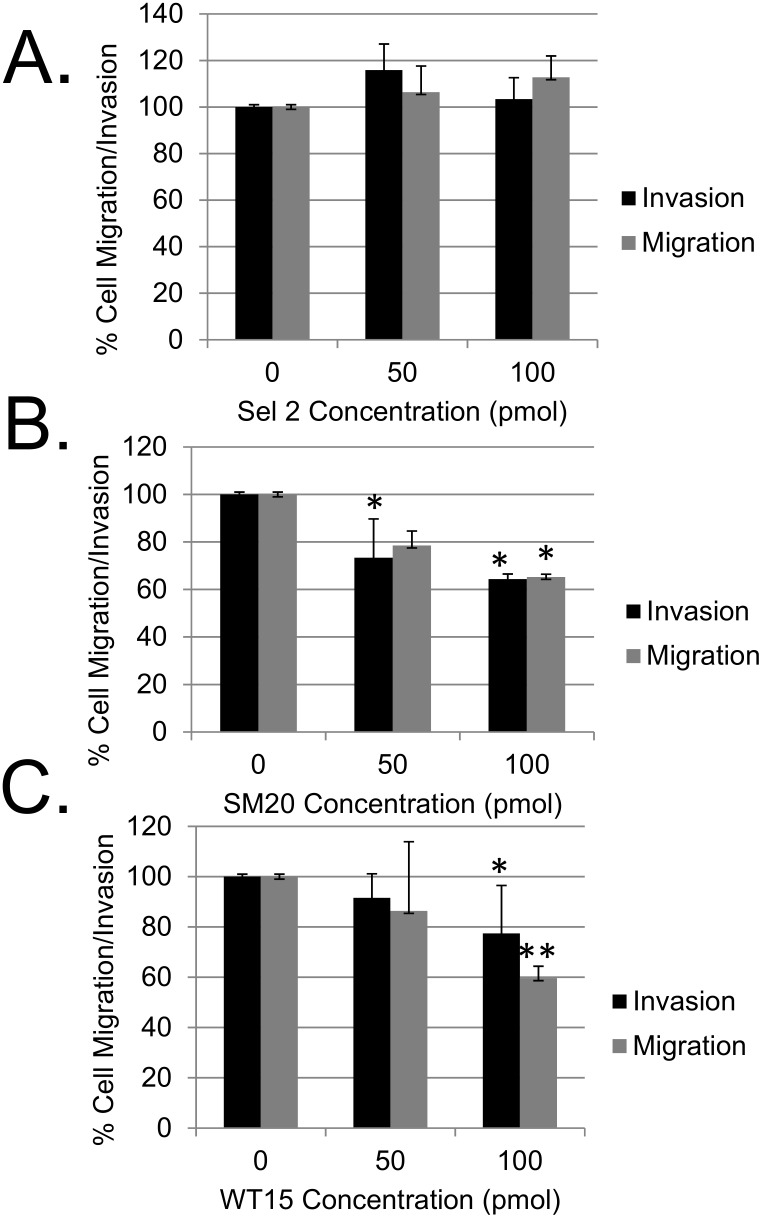
Effects of RNA aptamers on migration and invasion of MDA-MB-231 cells. MDA-MB-231 cells transfected with Sel2 (**A**), SM20 (**B**), and WT15 (**C**) were added to transwell inserts. For migration assays, the cells were added to uncoated transwell inserts and allowed to migrate for 18–24 hours at 37°C. For invasion assays, the cells were added to transwell inserts coated with Matrigel. The cells were allowed to invade for 24 hours at 37°C. Chemo attractants were added to the lower well. Results shown represent the average +S.D. from three independent assays that were performed in duplicate. All data were normalized to migration or invasion in non-transfected cells, which was set at 100%. *p<0.05 compared with PAI-1 alone. Each data point was performed in triplicates and the experiments were repeated at least three times with similar results. *p<0.05, **p<0.01.

Cell migration and invasion are both required for breast cancer metastasis. Consequently, we evaluated the ability of the transfected aptamers to inhibit migration and invasion of MDA-MB-231 breast cancer cells. Cells transfected with either SM20 or WT15 migrated slower when compared to both non-transfected cells and ones transfected with the control aptamer (Fig 4B and 4C). Likewise, fewer cells invaded as compared to non-transfected cells, with the largest overall effect seen in cells transfected with SM20. However, cells transfected with 100 pmol WT15 displayed more significant decrease in migration compared to non-transfected cells and ones cells transfected with SM20 (Fig 4B and 4C). The control aptamer did not cause a decrease in cell migration or invasion (Fig 4A). Both decrease in migration and invasion of MDA-MB-231 cells were concentration dependent **(**Fig 4B and 4C). The slight increase in cell migration in cells transfected with our control aptamer was not significant (Fig 4A). These data further support our hypothesis that PAI-1 is inhibiting uPA, causing a decrease in plasmin generation, which results in attenuated breast cancer cell migration and invasion.

### Transfection of aptamers into HUVECs

**Fig 5:**
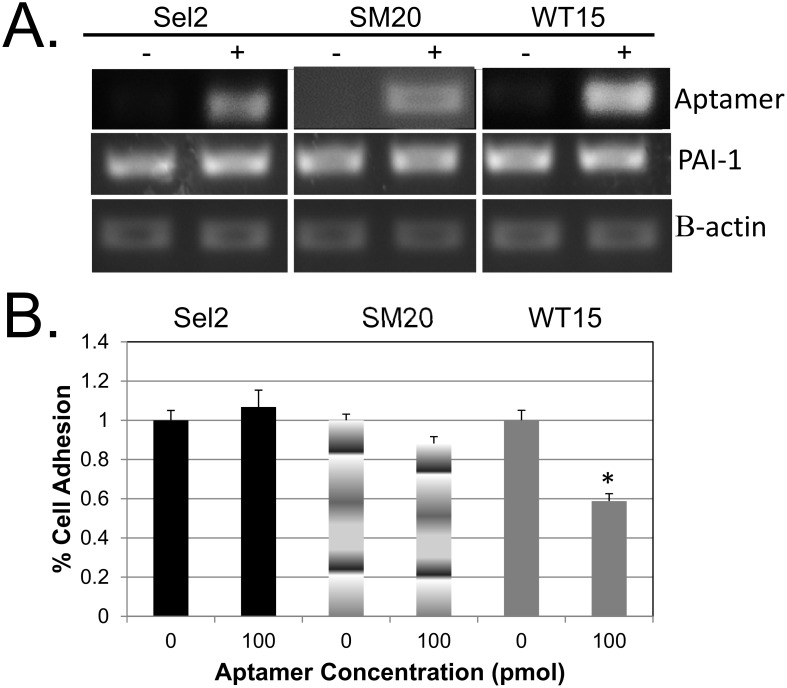
Expression of RNA aptamers in HUVECs. (**A**) Total RNA was isolated from transfected (+) and non-transfected (-) cells, then RT-PCR analysis were performed. Expression of the aptamer, PAI-1, and β-actin is shown. N.B. The SM20 was assay was run separately and then added to the figure. (**B**) HUVECs transfected with aptamers (Sel2, SM20, and WT15) or non-transfected cells were added to vitronectin coated plates and incubated for 1 hour at 37°C. The non-adherent cells were removed and the adherent cells were assessed by an MTT assay analysis. The percent of adherent cells were normalized to the percent of cells adhering in the absence of aptamers. All reactions were done in triplicates and repeated at least twice times; error bars represent the standard deviation of the data. *p<0.05.

Given the role that PAI-1 plays in regulating angiogenesis [25–27], we sought to determine the effect of the aptamers on tube formation in HUVECs by transiently transfecting them with our aptamers. Similar to the MDA-MB-231 cells, these aptamers were effectively transfected into the cells (Fig 5A). Also, similar to MDA-MB-231 cells, there was no significant change in PAI-1 expression (Fig 5A). The aptamers were not toxic to these cells, as both transfected and non-transfected cells looked healthy and cell viability was maintained (data not shown). Next we assessed the adhesive properties of the transfected cells. Cell adhesion of HUVECs transfected with WT15 was significantly decreased compared to non-transfected cells (Fig 5B). Thus, as in MDA-MB-231 cells, we observed a more profound effect on adhesion in cells transfected with WT15.

### Tube formation is disrupted in HUVECs transfected with the PAI-1 aptamers

**Fig 6:**
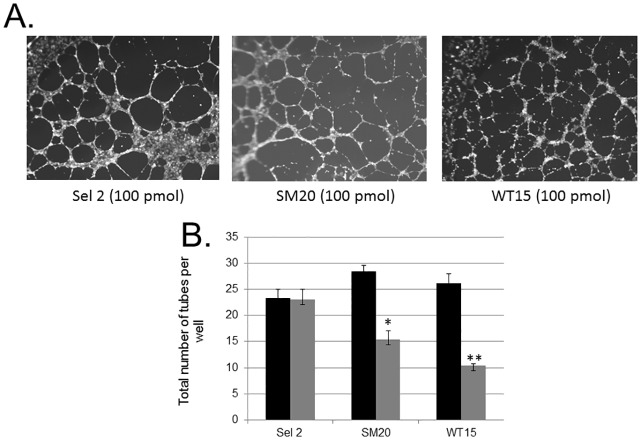
Transfected aptamers in HUVECs decrease tube formation. HUVECs were transfected with the various aptamers. Forty-eight hours post-transfection, the cells (1.5x10^4^) were placed on matrigel and incubated at 37°C. Tubes formed within 24 hours. The slides were photographed (**A**) and the total number of tubes was counted by a blinded mechanism (**B**). Data represent the average number of tubes formed per well from three independent experiments performed in duplicates. Error bars represent the standard deviation of the data. Representative photos are shown. *p<0.05, **p<0.01.

Next we evaluated the ability of transfected HUVECs to form tubes. A significant disruption of tube formation was detected in cells transfected with both SM20 and WT15 aptamer with the largest effect seen in cells transfected with WT15 (Fig 6A and 6B). There was no difference in the number of tubes formed in cells transfected with the control aptamer compared to non-transfected cells (Fig 6B). We also noted a change in the morphology of tubes formed in cells transfected with the experimental aptamers compared to the control aptamer, including the diameter of the tubes (Fig 6A). Collectively, these data imply that the aptamers are causing a decrease in the overall ability of the endothelial cells to form tubes, which indicates a decrease in angiogenesis or a potentially ‘anti-angiogenic effect’.

#### The cytokines secreted by transfected MDA-MB-231 cells has an effect on angiogenesis

**Fig 7:**
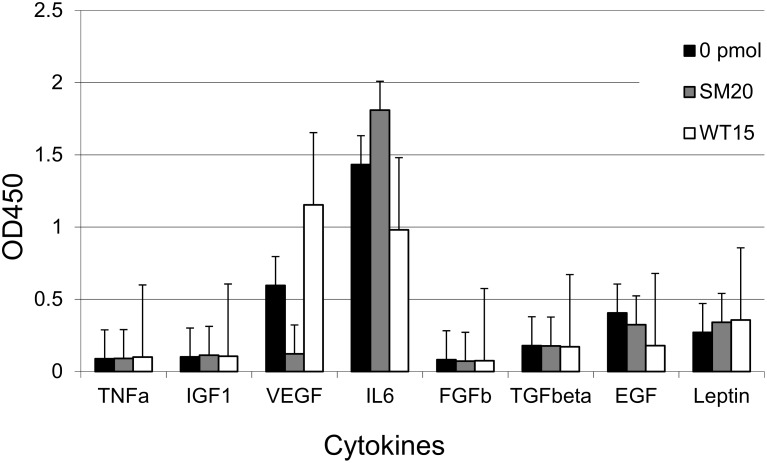
Levels of secreted cytokines in the conditioned medium of transfected and non-transfected cells. Conditioned medium from cells transfected with either SM20 or WT15 and non-transfected cells were collected and assayed for cytokines expression as detailed in Materials and Methods. Data represent the average of three to four independent transfection experiments. Error bars represent the standard deviation of the data.

Next, we determined if the cytokines secreted by the transfected MDA-MD-231 cells alter HUVEC tube formation. We analyzed the levels of the major cytokines in the conditioned medium from transfected and non-transfected cells and observed no change in TNFalpha, IGF1, FGFb or TGFβ. The levels of VEGF was increased in conditioned medium from cells transfected with WT15 and decreased in cells transfected with SM20. On the other hand, the IL6 expression was increased in cells transfected with SM20 but decreased in cells transfected with WT15. There was a slight decrease in EGF and a slight increase in leptin in response to both aptamer treatments (Fig 7).

**Fig 8:**
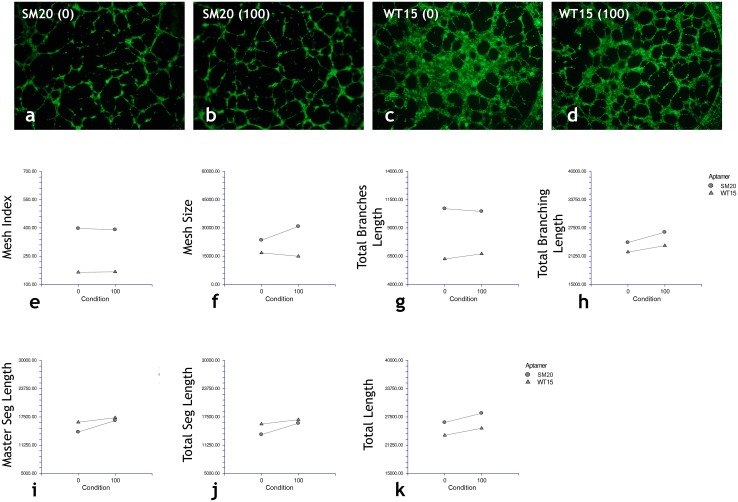
Cytokines secreted by transfected MDA-MB-231 cells have an effect on angiogenesis. Images taken at 4× magnification of calcein labeled tubes formed by HUVECs transfected with either (a, b) SM20 or WT15 (c, d) aptamer and grown in conditioned media from MDA-MB-231 cells. The number next to each aptamer type indicates the concentration of the aptamer (0 or 100 pM). (e-k) Morphological parameters assessed from images of the tube formation assay. Each plot indicates the difference in the parameter as a function of aptamer type (i.e. SM20 vs. WT15) or aptamer concentration (i.e. 0 vs. 100 pM).

The conditioned medium from aptamer transfected MDA-MB-231 cells was used on an in vitro HUVEC tube formation assay. Interestingly, the CM from the transfected MDA-MB-231 cells had a slight pro-angiogenic effect as determined by assessing various morphological parameters that describe the tubule network formed by HUVECs (Fig 8). The parameters for which both the aptamer type and concentration had a concurrent significant effect were the total branching length master segment length, total segment length and total length of the tubes (Fig 8h–8k). The type of aptamer had a significant effect on both the mesh index and total branches length (Fig 8e–8g). These results are summarized in Table 1.

**Table 1:** Summary of Morphological Data from HUVEC Tube Formation Assay.

Morphological Parameter	Results of Repeated Measures ANOVA Significant differences between aptamers (A), i.e. SM20 vs. WT15 or Condition (C), i.e. 0 pM vs. 100 pM.
MESH INDEX	**A: 0.0014**
	C: 0.9531
MEAN MESH SIZE	A: 0.1306
	C: 0.5166
TOTAL BRANCHES LENGTH	**A: 0.00003**
	C: 0.7975
TOTAL BRANCHING LENGTH	**A: 0.0201**
	**C: 0.0050**
TOTAL LENGTH	**A: 0.0025**
	**C: 0.0024**
TOTAL MASTER SEGMENT LENGTH	A: 0.2144
	**C: 0.0122**
TOTAL SEGMENT LENGTH	A: 0.1706
	**C: 0.0140**

## Discussion

Several studies have demonstrated that cancer cells produce a high level of endogenous PAI-1 [28–31]. Whereas PAI-1 is a secreted serpin, under pathological conditions, such as cancer, cell associated PAI-1 levels are increased both inside the cell and in the blood plasma [32]. Selectively inhibiting intracellular PAI-1 expression has been accomplished previously by siRNA or shRNA approaches [33–36]. However, these approaches inhibit the protein from being translated, resulting in a decrease in both RNA and protein expression. To the best of our knowledge, there have been no reports about the selective inhibition of the intracellular PAI-1 protein by RNA aptamers.

Aptamers are novel nucleic acid molecules that target intracellular and extracellular proteins and the number of inhibitory aptamers being developed as therapeutics is steadily growing [37,38]. In this study, we provide evidence that endogenously expressed aptamers exert biological effects on both cancer and endothelial cells. Our results show that PAI-1 specific aptamers inhibit the metastatic potential of breast cancer cells, in addition to inhibiting angiogenesis. Our major finding that the aptamers causes a decrease in uPA activity and an increase in the PAI-1/uPA complex imply that they are converting these highly invasive human breast cells to a less invasive phenotype. These data open up the possibility of the therapeutic use of aptamers in cancer treatment.

Indeed, numerous aptamers have been developed to target breast cancer cells. For example, cell-SELEX was used to identify aptamers that specifically bind to and recognize the MCF-10AT1 breast cancer cells [39]. Also, a more recent study identified several DNA aptamers that recognize MDA-MB-231 breast cancer cells [40]. Using cell SELEX, Zueva et al., identified one aptamer that bind bound to the surface of HET-SR-1 metastatic cells without being internalized and another that was internalized in these cells [41]. Both aptamers had an effect on cell migration and invasion [41]. Similar to our results, this study demonstrated that aptamers could alter the metastatic potential of cancer cells upon intracellular expression. The critical difference between the two studies is that our aptamers targeted a protein, PAI-1, that is known to have an effect on tumor cell migration, invasion and angiogenesis [9]. While we used a vehicle to express our aptamers in these cells, we showed that they significantly altered the metastatic potential of aggressive breast cancer cells. This is proof of principle that aptamers can have an endogenous effect on cancer cells.

Liposomes have been used to introduce aptamers into various cells either by incorporating the aptamers into expression vectors or via direct delivery [42,43]. In our studies we used the direct delivery approach. There are several acceptable methods for introducing nucleic acids into cells, including via nanoparticles or via binding to surface bound receptors. However, the ability of aptamers to target intracellular targets has proven to be a daunting task mainly due to insufficient delivery of cytosolic aptamers. The expression of intracellular aptamers is termed intramers. Blind et al., initially showed that cytoplasmic expression of intramers regulated integrin mediated cell adhesion [44]. Since then, follow up studies have shown expression of intramers in various cells [44,45]. More recently, Liu et al., demonstrated the intracellular expression of an aptamer to EGFRvIII which interacts with newly synthesized the EGFRvIII protein [46]. Also, the intracellular expression of aptamers to PPAR specific aptamers was shown to decrease the tumorigenic potential of colon cancer cells [47]. In each of these studies the aptamers (intramers) were transfected directly into the cells. Very few aptamers are directly taken up by cells without the aid of vectors or other vehicles. However, a recent approach termed, “cell internalization SELEX” [39,48,49] is able to achieve this. In this approach, the aptamers are incubated with the whole cell; however, instead of selecting for molecules that bind to the surface, molecules that are shuttled into the cells are selected [48–50]. The aptamers are not targeted to a specific protein but are instead selected against the entire cell. The aptamers bind to cell surface receptors or surface proteins, and are then internalized. Several groups have shown this particularly in HPV transformed cells [51], in cells expressing PMSA [52,53], and in acute leukemia cells [48]. Generally, the mechanism by how this occurs is unknown and the target protein or receptor is also unknown. Aptamers have also been used for delivering nucleic acid therapeutics such as siRNAs into cells via siRNA-aptamer chimeras [52], but studies investigating the action of aptamers inside the cell are lacking. Our aptamers were utilized, not as delivery agents, but instead as functional molecules inside breast cancer and endothelial cells. Our study shows that expressing functional aptamers inside breast and endothelial cells is feasible and they also exhibit therapeutic potential. These findings open up the possibility of aptamer-aptamer chimeras, wherein one aptamer serves as the delivery molecule while the other functions as the therapeutic agent.

Generally, aptamers bind to their target protein, resulting in either inhibition or in some cases, enhancement of the protein’s function [16,19,54]. Inhibition is usually via a direct effect; however, it can also be indirect. For example, altering the target protein from binding to its target substrate could inhibit the activity of downstream effectors, as has been shown in interleukin signaling [55]. PAI-1 has been shown to promote and inhibit these processes via its ability to inhibit plasmin generation and by binding to vitronectin [56–59]. We reported previously that exogenously adding the aptamers and PAI-1 to MDA-MB-231 cells, resulted in an increase in cell migration [20]. This was most likely caused by the binding of the aptamer to the vitronectin binding site of PAI-1 [20,22]. Thus, exogenous PAI-1-mediated cell migration is abrogated by these aptamers [22]. Despite the exogenous aptamer data, when these aptamers were expressed inside the cell we observed the opposite effect. Indeed the finding that the invasiveness of breast cancer is correlated with high PAI-1 expression has long puzzled investigators [1,60]. PAI-1 which inhibits uPA should inhibit cancer progression. A partial explanation for this paradox is based on the finding that PAI-1 interacts with several molecules and exerts proteolytic and non-proteolytic activity such as intergins and vitronectin [61–63]. Nevertheless, the ‘paradox’ remains under investigation. Consequently, several laboratories have been activity developing small molecule PAI-1 inhibitors [14]. We propose that the explanation could lay in the mechanism by which PAI-1 interacts endogenously. The aptamer dependent decrease in cell migration and invasion appears to be partly due to the PAI-1’s inhibitory potential, as uPA activity is decreased both intracellularly and extracellularly, as we showed a decrease in secreted uPA activity. These data are in contrast to data by which PAI-1 expression was decreased by siRNA [31]. MDA-MB-231 cells transfected with PAI-1 siRNA showed a significant decrease in PAI-1 protein that correlated with a protective effect on cancer cell apoptosis [31]. Others have reported that inhibiting PAI-1 by using siRNA techniques decreased PAI-1 and tumor cell growth [35,64]. Whereas, we likewise showed a decrease in PAI-1 protein, no change in mRNA levels was detected. Instead, the decrease in protein was due to an increase in PAI-1’s interaction with uPA. Hence, these results suggest that the aptamers, either because of their ability to alter PA-I-1’s interaction with other proteins or because of a conformational change induced by the aptamers, increased PAI-1’s inhibitory potential towards uPA. Decreases in unbound “free” PAI-1 protein were not due to a classical inhibition of the protein, but instead it is due to an increase in the interaction of PAI-1 with uPA.

PAI-1’s role in angiogenesis is likewise complex. Physiologic levels of PAI-1 promote angiogenesis [11,65], while pharmacological levels inhibit angiogenesis [66]. Interestingly, we provide data suggesting that the PAI-1 specific aptamers are directly able to inhibit PAI-1 mediated angiogenic potential. However, when we incubated HUVECs with conditioned media from aptamer transfected MDA-MB-231 cells, we observed a slight proangiogenic effect. It is well known that MDA-MB-231 cells secrete high levels of pro-angiogenic growth factors such as vascular endothelial growth factor (VEGF) amongst others [67], therefore it is possible that this pro-angiogenic cytokine soup overwhelms any anti-angiogenic effects of the endogenous aptamers, thereby promoting tube formation. Interestingly, VEGF was increased and IL6 decreased in conditioned media from cells transfected with WT15, which could potentially account for the slight pro-angiogenic effect. However, the opposite was observed formedia secreted from cells transfected with SM20. Additional studies are planned to answer questions such as whether higher levels of aptamer transfection are necessary for exercising an antiangiogenic effect or is there another mechanism in the PAI-1/uPA pathway by which this may occur.

While targeting PAI-1 as a therapeutic option for cancer treatment has gained attention over the years, it is a fairly new area. Although, the potential of using PAI-1 inhibitors in cancer therapy is possible, there are still several challenges [68]. This study suggests that using aptamers that target PAI-1 as inhibitors can lead to future molecules that can be used in cancer therapies affecting multiple hallmarks of cancer, such as invasion, migration and angiogenesis [69]. Additionally, these molecules are not restricted to the extracellular compartment but may also be viable intracellular therapeutic agents, as well.

## Supporting Information

S1 Fig (a) Terms defining the network topology. Image taken at 4× magnification of calcein labeled tubes formed by HUVECs overlaid with the output of the ImageJ Angiogenesis Analyzer plugin. (b) Pooled results of the effect of each aptamer on angiogenesis assessed via the morphological parameters extracted from the tube formation assay images. Each plot indicates the trend in the parameter as a function of aptamer type (i.e. SM20 vs. WT15) or aptamer concentration. This plot is for illustrative purposes only and was not subjected to statistical analysis because the 0 and 100 μM samples were pooled.
(TIF)
