# Supplementary figures and images for: Intracellular Expression of PAI-1 Specific Aptamers Alters Breast Cancer Cell Migration, Invasion and Angiogenesis
Source: PLoS One. 2016 Oct 18;11(10):e0164288. doi: 10.1371/journal.pone.0164288 (PMC5068744; doi:10.1371/journal.pone.0164288)

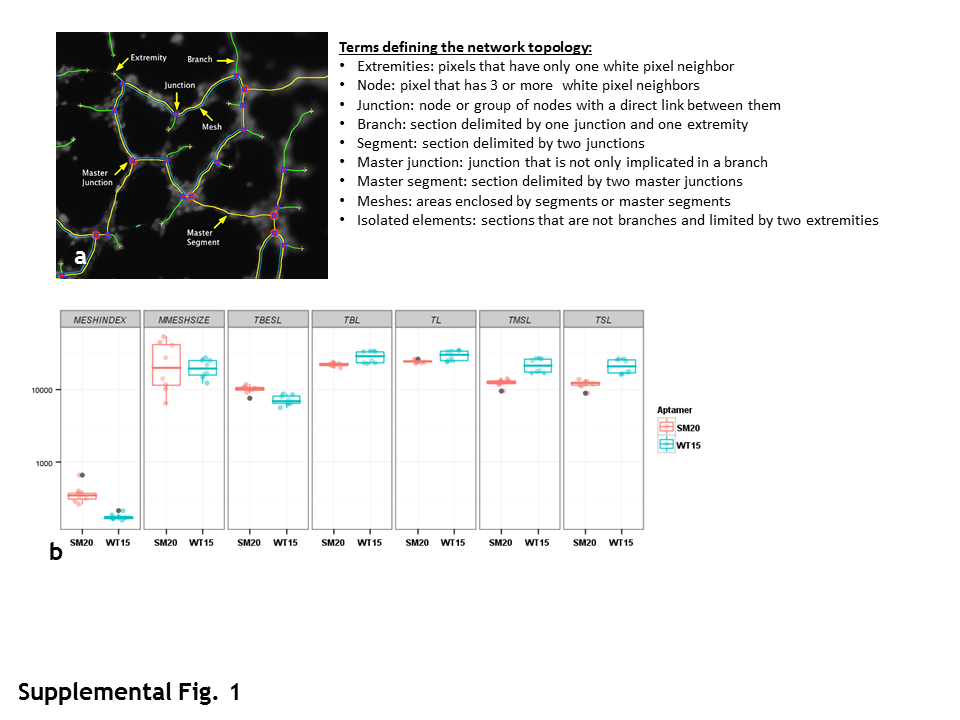

Supplement: S1 Fig — (a) Terms defining the network topology. Image taken at 4× magnification of calcein labeled tubes formed by HUVECs overlaid with the output of the ImageJ Angiogenesis Analyzer plugin. (b) Pooled results of the effect of each aptamer on angiogenesis assessed via the morphological parameters extracted from the tube formation assay images. Each plot indicates the trend in the parameter as a function of aptamer type (i.e. SM20 vs. WT15) or aptamer concentration. This plot is for illustrative purposes only and was not subjected to statistical analysis because the 0 and 100 μM samples were pooled. (TIF) [file pone.0164288.s001.tif]
